# Life-threatening hepatic complications in children with ADA-SCID

**DOI:** 10.70962/jhi.20260112

**Published:** 2026-09-30

**Authors:** Ridhima Aggarwal, Alison Mansfield, Jinhua Xu-Bayford, Claire Booth, Konstantinos Vazouras

**Affiliations:** 1 University College London Great Ormond Street Institute of Child Health, London, UK; 2 Department of Immunology and Gene Therapy, Great Ormond Street Hospital for Children NHS Foundation Trust, London, UK

## Abstract

We describe two cases of ADA-SCID complicated by hepatoblastoma and infantile hepatic hemangiomatosis. We highlight the multidisciplinary management of these severe liver manifestations and the modifications required to deliver definitive corrective therapy, including gene therapy and hematopoietic stem cell transplantation.

## Teaser

We report two cases of ADA-SCID complicated by severe hepatic manifestations, including hepatoblastoma and infantile hepatic hemangiomatosis. These cases highlight the challenges of multidisciplinary management and the need to modify definitive corrective therapies, including gene therapy and hematopoietic stem cell transplantation, in children with complex liver disease.

## Introduction

Adenosine deaminase (ADA) is a universally expressed enzyme crucial for detoxification of cytotoxic purine metabolites. ADA deficiency classically results in life-threatening severe combined immunodeficiency (SCID) due to lymphopenia of T, B, and natural killer (NK) cells. In addition, due to the ubiquitous nature of ADA, nonimmunologic manifestations involving multiple systems are seen including neurodevelopmental, urogenital, hearing, and, albeit lesser reported, hepatic manifestations (1). Here, we describe two cases of ADA-SCID with life-threatening liver complications that delayed administration of corrective therapy. We also present a literature search of previously reported noninfectious, non–therapy-related hepatic complications in ADA-SCID.

## Patient 1

A full-term male infant was the third-born child to a nonconsanguineous Caucasian couple. He was referred to our center at 3 mo of age with COVID-positive bronchiolitis, chronic (vaccine strain) rotavirus gastroenteritis, oral thrush, and failure to thrive. Full blood count showed lymphopenia (absolute lymphocyte count: 0.24 × 10^9^/liter) and thrombocytosis (1,027 × 10^9^/liter). Lymphocyte subset analysis revealed severely reduced T cells (0.01 × 10^9^/liter), B cells (0.03 × 10^9^/liter), NK cells (0.15 × 10^9^/liter), and absent T cell receptor excision circles (0 copies). IgM and IgA were undetectable, and IgG was reduced (1.9 g/liter). ADA activity was 0 nmol/mgHb/h (normal: 40–100 nmol/mgHb/h), and deoxyadenosine triphosphate (dATP) levels were elevated (223 µmol/liter, normal: undetectable). Next-generation sequencing identified a compound-heterozygous variant in *ADA*: c.424C>T, p.Arg142* and deletion of exon 1.

He was acutely managed with supplemental oxygen, antivirals (remdesivir and nitazoxanide—an antiparasitic agent repurposed for COVID-19 treatment), and antibiotics (piperacillin/tazobactam, ciprofloxacin, clarithromycin, and nystatin). He was initiated on prophylactic antibiotics (cotrimoxazole and fluconazole), immunoglobulin replacement therapy (IgRT), and PEGylated recombinant ADA enzyme replacement therapy (ERT). As a matched donor could not be identified, he was referred for lentiviral gene therapy (GT). He underwent peripheral stem cell mobilization and apheresis at 9 mo of age, with a plan to administer GT within 2 mo.

However, at 11 mo of age, he was found to have new-onset hepatomegaly and weight loss. Blood tests revealed alanine aminotransferase (ALT) of 158 U/liter and α-fetoprotein of 36 kU/liter. Abdominal ultrasound revealed a hypoechoic nodule in segment V of liver, measuring 6 × 6 × 6 mm, which was then biopsied. Histopathologic examination showed atypical hepatocytes (positive histochemistry for glypican-3, MNF116, and CD99) in keeping with possible hepatoblastoma. There were no histological signs of fungal infection, but *Aspergillus* sp. was isolated on initial fungal culture and repeat subculture. A plan was made to proceed with surgical resection and treatment with liposomal amphotericin B.

He underwent a restricted segmental V liver resection at 13 mo of age; histology confirmed epithelial-type hepatoblastoma without vascular invasion. He received adjuvant chemotherapy with two cycles of low-dose cisplatin 21 days apart. He tolerated chemotherapy well, and ALT subsequently normalized. Magnetic resonance imaging confirmed resolution but showed a new faint 4-mm lesion in segment VIII, which stabilized 6 wk after chemotherapy. At 18 mo of age, he underwent reduced-intensity conditioning with busulfan (area under the curve target 20 mg*h/liter) followed by infusion of autologous cryopreserved gene-corrected CD34^+^ cells (transduced with a self-inactivating lentiviral vector containing the *ADA* driven by the EF1α short promoter).

At 4 years of age, he has had excellent cellular immune reconstitution (T cells 1.25 × 10^9^/liter, B cells 0.59 × 10^9^/liter, and NK cells 0.31 × 10^9^/liter), normal immunoglobulin levels, restored ADA activity, and stable gene marking. The segment VIII liver lesion became undetectable by 3 years of age, and he has remained well off ERT, IgRT, and prophylactic antibiotics.

## Patient 2

A full-term female child, born to a third-degree consanguineous couple of Pakistani origin, was symptomatic from day 7 of life with respiratory failure, late-onset sepsis, and rapidly progressive hepatomegaly. Further evaluation revealed thrombocytopenia and lymphopenia. Lymphocyte subsets showed absent T, B, and NK cells, absent proliferation response to phytohemagglutinin, absent IgM, and IgG of 2.94 g/liter. ADA activity was reduced (18 nmol/mgHb/h, normal: 40–100), and dATP levels were elevated (590 umol/liter, normal: undetectable). Whole-exome sequencing revealed a homozygous pathogenic *ADA* variant; c.646G>A, (p.Gly216Arg), previously described in patients with ADA deficiency. She was treated with broad-spectrum antibiotics (piperacillin/tazobactam and amikacin) for presumed bacterial infection, while cotrimoxazole, fluconazole, and ganciclovir were administered as antimicrobial prophylaxis.

Ultrasound abdomen revealed a large, diffusely heterogeneous liver with multifocal patchy areas of low and high echogenicity consistent with diffuse infantile hepatic hemangiomatosis (IHH). Biopsy from a nodule in the left lobe revealed irregular vascular channels confirming diagnosis. Her clinical condition deteriorated requiring mechanical ventilation with two episodes of cardiac arrest related to hemodynamic instability caused by the giant hemangiomas. Weekly vincristine (25 microg/kg, 8 doses) was initiated to treat the hemangiomas alongside PEGylated recombinant ADA ERT and IgRT. After clinical stabilization, prednisolone was added (2 mg/kg/day for 4 wk followed by tapering). She was discharged at 3 mo of age, and serial ultrasounds showed the lesions reducing in number and echogenicity and ultimately becoming calcified.

At 9 mo of age, a recurrence occurred with an increasing number of lesions in liver, the largest measuring 1.7 cm. New-onset superficial hemangiomas were noted on her back, and prednisolone (2 mg/kg/day) was reinitiated. However, within weeks, she became symptomatic with rotavirus gastroenteritis diarrhea, adenoviremia, and parainfluenza type 3 in her nasopharyngeal aspirate. She was initiated on cidofovir and ribavirin but remained symptomatic. She subsequently became positive for influenza B and received oseltamivir.

In view of multiple viral infections, prednisolone was stopped, and vincristine was reinitiated and continued until hematopoietic stem cell transplantation. She was conditioned with treosulfan 36 g/m² and cyclophosphamide 200 mg/kg, and received a 6/6 matched unrelated cord blood transplant (cell dose: 12 × 10^7^/kg) at the age of 12 mo.

Serial abdominal ultrasounds after transplant showed resolution of hepatic lesions, which became difficult to visualize individually. Immune reconstitution was complete and sustained. Last seen at 18 years of age, she remains 100% donor engrafted, with no infections, autoimmunity, or malignancy.

## Discussion

To the best of our knowledge, this is the first case series focusing on the management of liver tumors prior to corrective therapy for ADA-SCID. Hepatoblastomas are rare pediatric tumors occurring in children in first 3 years of life, with the most common risk factor identified being very low birth weight (2). This tumor arises from fetal multipotent progenitor cells that have been exposed to local cytokines resulting in differentiation into several lineages. Other liver tumors—namely, hepatocellular carcinoma, hepatic adenoma—have also been previously reported in ADA-SCID (3). It has been postulated these may occur as a result of accumulation of toxic metabolites causing an increase in bile salt–mediated chronic inflammation leading to metaplasia (3). In our case, the child was detoxified early after diagnosis at 2 mo of age following initiation of ERT, making a link with toxic metabolites unclear. While reduced tumor surveillance due to absent or dysfunctional T cells remains a possibility for susceptibility to malignancy, ADA-SCID as a risk factor for increased incidence of hepatic malignant tumors remains to be studied. In this case, we also highlight key advantages of the use of GT—including ability to cryopreserve transduced stem cells that allowed for flexibility to delay corrective therapy, improved liver toxicity profile with low-dose busulfan conditioning, and absence of liver graft-versus-host disease (GVHD).

IHH is the most common benign liver tumor in infancy that presents at birth, proliferates until 6–12 mo, and involutes over a few years (4). Diffuse IHHs, though benign, may present with life-threatening complications such as rapidly progressive hepatomegaly and cardiorespiratory failure. Pathogenesis of IHH has been suggested to be linked to embolization of placental cells or a shared developmental pathway to placenta as they share the same endothelial markers (GLUT1) (4). The growth and proliferation of endothelial cells are thought to result from elevated local concentrations of vasculogenic factors. It seems unlikely that an accumulation of toxic metabolites significantly contributed to the occurrence or rapid progression of diffuse IHH in our patient.

Of note in this case, the use of steroids was restricted due to underlying immunodeficiency, while severity of clinical features prompted the use of vincristine as a safer alternative. Here, we emphasize the use of ERT as a safe, detoxifying bridge therapy that allowed for use of chemotherapy specific to IHH and time for resolution. This is the only report of a life-threatening IHH occurring concurrently in a case of ADA-SCID that required aggressive medical management; however, the true incidence of hemangiomas in ADA-SCID is unknown as many lesions are asymptomatic and stay unidentified.

In ADA-SCID patients, liver disease is more commonly attributed to complications arising due to immune deficiency or its treatment, such as infections, hepatotoxic medications, and GVHD. Metabolic hepatopathy, immune dysregulatory, and neoplastic hepatic complications in ADA-SCID are rarely reported (Table 1). In keeping with previous literature, hepatic steatosis, noninfectious transaminitis, focal nodular hyperplasia, and nodular regenerative hyperplasia have also been seen in some ADA-SCID patients in our cohort. Clinicians involved in the care of ADA-SCID patients should maintain a high index of suspicion for serious noninfectious hepatic complications, as prompt recognition and management are critical to optimizing long-term outcomes.

**Table 1.** Review of literature on liver manifestations seen in ADA-deficient SCID

Study	Liver condition	Immunogenetic status	Histopathology and biopsy testing	Laboratory and imaging	Treatments and outcomes
**Noninfectious hepatitis/hepatopathy**					
Bollinger et al. (1996)	Metabolic hepatopathy Presentation: hepatomegaly and jaundice (3 wk)	Genetics: compound heterozygous (*ADA*: c.316G>T; c.1081C>T) Immune status ADA activity^a^: 0 dATP^a^: 709 nmol/ml of packed cells (*N* < 2) Hypogammaglobulinemia Severe lymphopenia	Histopathology: Giant cell transformation Enlarged foamy hepatocytes, portal and lobular infiltrates No evidence of transplacental maternal engraftment. No viral inclusions Biopsy testing: Stain for HSV/CMV: negative Viral culture of liver: negative	Biochemistry: Total Bil: 5 mg/dl D. Bil: 2.9 mg/dl AST/ALT: 561/109 IU/liter ALP: 528 IU/liter LDH: 1997 IU/liter Blood investigations: Bacterial cx: negative Viral cx: negative CMV/EBV/Hep A/Hep B: serology negative PCR Hep C: negative	ERT with PEG-ADA Rapid improvement in bilirubin and transaminases prior to T cell recovery At 23 m: lymphocyte proliferation and response to tetanus toxoid normal
Kühl et al. (2011)	Hyperbilirubinemia and rapid hepatic failure Presentation: neonatal jaundice Transaminitis	Genetics: homozygous (*ADA*: c.632 G > A) Immune status: ADA activity: 30 IU (160–1,030) Lymphopenia: 0.133 × 10^9^/liter Low T cells: 0.044 × 10^9^/liter B cells: 0.001 × 10^9^/liter NK cells: 0.022 × 10^9^/liter Lymphocyte proliferation absent with mitogen TCRV β repertoire of both peripheral CD4^+^ and CD8^+^ T cells—markedly restricted	​	Biochemistry AST/ALT—999/271 IU/liter Blood investigations CMV/EBV/HSV1/HSV2/HHV6/HHV7/ADV/enterovirus/HIV1/2/parvovirus B19 by PCR—negative HAV, HBV, HBC, HDV, and HEV: PCR from serum negative	ERT MRD 10/10 (HLA identical father) Empiric antibiotics Did not survive
Somech et al. (2009)	Autoimmune hepatitis Presentation: transaminitis (19 m) Comorbidities: inflammatory pneumonitis (18 m)	Genetics: compound heterozygous (*ADA*: c.7C>T; c.529G>A) Immune status: ADA activity: 3% of normal control values Reduced CD3, CD19, and CD56 lymphocyte responses to mitogens—reduced TREC: 740 copies per 0.5 µg of DNA (like healthy) T cell repertoire: normal representation of all 23 Vb groups tested	Histopathology: Prominent inflammatory cell infiltrates in portal tracts extending into adjacent peripheral parenchyma Foci of piecemeal necrosis Increased portal and periportal fibrous tissue with some bridging fibrosis Biopsy testing: Stain for adenovirus/HSV 1 and 2/CMV/EBV: negative	Biochemistry ALT—2653 IU/liter Autoantibodies Anti-liver and anti-kidney microsomal antibody levels (+ at 1:640) Anti-SMA (+ at 1:32) Anti-tTGA—positive (31 U/ml)	ERT with PEG-ADA No improvement in ALT despite ERT for 6 mo Prednisolone 1 mg/kg/day Complete resolution in 1 m
Vittal et al. (2023)	Hepatic steatosis (*n* = 6) Presentation: asymptomatic	Genetics: N/A	Histopathology Mild-to-moderate macrovesicular steatohepatitis with mild inflammation predominantly in Zone 1	Hepatic USG showed steatosis; *n* = 10	Screened 18 ADA-SCID patients All received ERT GT *n* = 7 HSCT *n* = 5 No intervention for hepatic manifestation
Vittal et al. (2023)	Fibrosis (*n* = 3)	Genetics: N/A	Histopathology: P1: periportal fibrosis with perisinusoidal fibrosis P2: advanced bridging fibrosis	Hepatic USG showed steatosis; *n* = 10	Screened 18 ADA-SCID patients All received ERT GT *n* = 7 HSCT *n* = 5 No intervention for hepatic manifestation
**Hepatic tumors**					
Ucku et al. (2023)	Hepatocellular carcinoma Presentation: jaundice and hepatomegaly Comorbidities: GVHD	Genetics: c.792>A, p.(Trp264), homozygous Immune status 5.12 × 10^9^/liter Lymphocytes 0.6% CD19^+^ 0% CD3^+^ 14.7%	Histopathology 1 (at 6 m of age): Ductular proliferation, mixed portal inflammation, ductular cholestasis, and portal–periportal fibrosis Biopsy testing 1: Chronic GVHD excluded, infectious workup negative Histopathology 2 (at 12 m of age): Liver pathology examination (specimen from liver transplant)—nodular HCC—trabecular, well differentiated	Liver USG: 9–11-mm hyperechogenic lesion in the right lobe MRCP: chronic parenchymal changes and periportal thickening without sclerosing cholangitis	HSCT (at 2 m of age): MSD, no conditioning with GVHD prophylaxis Mesenchymal stem cell transfusions 2^a^
					Living donor liver transplant (at 10 m after HSCT) Clinically well in follow-up Maintaining full T cell chimerism No recurrence of HCC
Vittal et al. (2023)	Hepatoblastoma	Genetics: N/A	Hepatoblastoma; *n* = 1	N/A	Screened 18 ADA-SCID patients All received ERT GT *n* = 7 HSCT *n* = 5 Surgical resection of hepatoblastoma
Grunebaum et al. (2019)	EBV negative diffuse large B cell lymphoma Presentation: on ERT, weight loss, vomiting Comorbidities: developmental delay B/L SNHL GBS	Genetics: homozygous (*ADA*; c.7 C>T) Immune status Undetectable ADA activity Severe lymphopenia Hypogammaglobulinemia	Biopsy: EBV negative diffuse large B cell lymphoma, with cells characterized as CD20^+^CD10^+^CD30^+^BCL6^+^, EBER negative, and with high Ki67 proliferative activity	MRI: multiple soft tissue masses, the largest being 9.5 × 7.2 × 7.3 cm with mass effect on the adjacent hepatic veins, portal veins, and biliary tree PET: high metabolic activity within the liver mass and surrounding lymph nodes, consistent with lymphoma	ERT (CHOP-R)
Resnick and Kozakewich (1995)	Hepatic adenoma Presentation: found at autopsy	N/A	Biopsy: well circumscribed and isolated from surrounding liver parenchyma by a well-formed continuous fibrous capsule. No cirrhosis. No aplasia	N/A	N/A

ADA, adenosine deaminase; ADV, adenovirus; ALP, alkaline phosphatase; ALT, alanine aminotransferase; AST, aspartate aminotransferase; B/L, bilateral; Bil, bilirubin; CHOP-R, cyclophosphamide, doxorubicin, vincristine, prednisone, rituximab; CMV, cytomegalovirus; cx, culture; D. Bil, direct bilirubin; dATP, deoxyadenosine triphosphate; EBER, EBV-encoded small RNA; EBV, Epstein–Barr virus; ERT, enzyme replacement therapy; GBS, Guillain–Barré syndrome [verify meaning in Grunebaum]; GT, gene therapy; GVHD, graft-versus-host disease; HAV/HBV/HCV/HDV/HEV, hepatitis A/B/C/D/E virus; HCC, hepatocellular carcinoma; Hep, hepatitis; HHV, human herpesvirus; HIV, human immunodeficiency virus; HLA, human leukocyte antigen; HSCT, hematopoietic stem cell transplantation; HSV, herpes simplex virus; IU, international units; LDH, lactate dehydrogenase; m, month; MRCP, magnetic resonance cholangiopancreatography; MRD, matched related donor; MRI, magnetic resonance imaging; MSD, matched sibling donor; N, normal; N/A, not available; P1/P2, patient 1/2; PCR, polymerase chain reaction; PEG-ADA, polyethylene glycol–conjugated ADA; PET, positron emission tomography; SCID, severe combined immunodeficiency; SMA, smooth muscle antibody; SNHL, sensorineural hearing loss; TCR, T cell receptor; TREC, T cell receptor excision circle; tTGA, anti-tissue transglutaminase antibody; USG, ultrasound.

a ADA activity.

## Informed consent

Written informed consent for publication was obtained from the patient and/or their parents as appropriate, for the publication of the clinical information presented in this manuscript. All information has been anonymized to protect patient confidentiality.
